# The association between gestational body mass index change, cesarean section, and other pregnancy outcomes: Research protocol: Retrospective cohort analysis

**DOI:** 10.1016/j.mex.2025.103699

**Published:** 2025-10-29

**Authors:** Kirsten I Black, Owen Streeter, Arianne Sweeting, Pejman Adily, Mark Lauer, Adrienne Gordon, Vincenzo Berghella, Belinda R Bruce, Lauren Ferris, Bradley de Vries

**Affiliations:** a Reproduction and Perinatal Centre, Faculty of Medicine and Health, University of Sydney, Sydney, Australia; b Royal Prince Alfred Hospital Women and Babies, Sydney, Australia; c Sydney Institute for Women, Children and their Families, Sydney Local Health District, Sydney, Australia; d Sidney Kimmel Medical College of Thomas Jefferson University, Philadelphia, PA, USA; e Susan Wakil School of Nursing and Midwifery, University of Sydney, Sydney, Australia

**Keywords:** Gestational weight gain, Body mass index, Maternal outcomes

## Abstract

Increasingly the value of gestational weight gain (GWG) as a useful measure to predict obstetric outcomes including cesarean section is being questioned. We will undertake a retrospective cohort study of recorded births in 2023 from 24 to 42 weeks’ gestation for women aged from 16 to 49 years in the United States. Using multivariable Robust Poisson Regression, maternal body mass index (BMI) and BMI gain will be modelled on the primary outcome of cesarean delivery. The primary analysis will be restricted to nulliparous women if there is interaction between parity and BMI or BMI gain. A sample of 716,392 births will be required to show approximately a one percentage point increase in cesarean section for each BMI classification compared with normal BMI (18.5 to <25) or per unit gain in maternal BMI. We hypothesize that the risk of cesarean section will increase with an increase in the BMI category and that BMI at birth may be a more relevant predictor of cesarean section than pre-pregnancy BMI. This question is important to answer given recent evidence casting doubt on the association between excessive GWG, which has been the focus of most interventions (with modest impact at best), and adverse pregnancy outcomes.

## Graphical abstract

**Figure:**
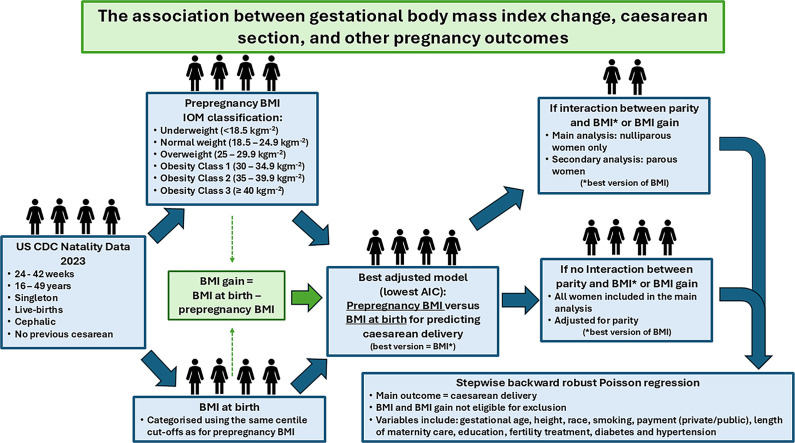

## Specifications table

**Table:** 

**Subject area**	Medicine and Dentistry
**More specific subject area**	Maternal obesity
**Name of your protocol**	The association between gestational body mass index change, cesarean section, and other pregnancy outcomes: Research Protocol: Retrospective Cohort Analysis
**Reagents/tools**	N/A
**Experimental design**	Retrospective cohort study
**Trial registration**	*N/A*
**Ethics**	Ethics approval was not required nor sought from any ethical committee, as publicly available de-identified data were used.
**Value of the Protocol**	*This protocol*

## Background

As of 2021, the worldwide average cesarean section rate was 21 %, and is predicted to increase to 30 % by 2030 [1]. In 2022, 39 % of babies in Australia and 32.1 % in the United States (US) were born by cesarean delivery [2,3]. Occurring in parallel is the obesity epidemic, with 61 % and 69 % of women in Australia and the US, respectively, living with overweight or obesity [4]. Obesity is a major risk factor for cesarean section, and higher risk of complications of the surgery including wound infection and longer hospital stay [5,6]. Cesarean delivery is in turn associated with greater risk for mothers and babies including maternal death, severe postpartum haemorrhage, endometritis [7,8], as well as complications for future pregnancies, such as preterm birth and abnormal placentation [9].

Interventions to reduce perinatal complications have focused on gestational weight gain (GWG), with studies linking it to adverse pregnancy outcomes, including cesarean delivery [[10], [11], [12]]. Excessive GWG is defined according to prepregnancy body mass index (BMI) by both the National Academy of Medicine (NAM) and the World Health Organization with lower GWG recommended for those starting pregnancy with a BMI in the obese category compared to those with normal BMI at the start of pregnancy [13,14]. Studies to date have included adjustment for pre-pregnancy or early pregnancy BMI, but, it is unknown whether this association could in fact be mediated by increased BMI at the time of delivery. Recently there has been some questioning of the value of GWG as a useful measure to predict obstetric outcomes. In a study by Dodd et al that used data from 20 randomized controlled trials, the authors identified a meaningful relationship between maternal BMI and GWG, whereby GWG decreased with increasing BMI. They also demonstrated a relationship between BMI and adverse pregnancy outcomes, whereby the risk of adverse pregnancy outcomes increased with increasing maternal BMI. However, they found no evidence that the effect of maternal BMI on outcomes is via an effect on GWG. That is, gestational weight gain may not be associated with cesarean delivery independently of BMI at the time of birth [16].

Given that finding, in this research we sought to explore whether using gestational BMI change is a better measure to use in understanding adverse obstetric outcomes. The primary objective of this study is to describe the relationship between gestational BMI gain and cesarean delivery and to compare pre-pregnancy BMI versus BMI at the time of birth as predictors of cesarean delivery.

## Description of protocol

### Method

**Study Design:** This will be a retrospective cohort study examining the relationship between gestational BMI change and the rate of cesarean section. The study will be reported according to STROBE Guidelines[17].

**Study Setting:** Eligible births recorded by the Center for Disease Control and Prevention 2023 Natality File, USA.

### Participants

#### Inclusion criteria

Inclusion criteria will include:

- Registered births in the United States in 2023 at 24 to 42 weeks’ gestational age at birth

#### Exclusion criteria

Exclusion criteria will include:

- Stillbirths (as the stillbirth dataset does not include maternal weight at delivery)
- Multiple pregnancies
- Non-cephalic presentations
- Previous cesarean delivery
- Maternal age < 16 years or maternal age > 49 years

### Primary study factors

- Change in maternal BMI from pre-pregnancy to birth
- Maternal BMI (either pre-pregnancy BMI or BMI at birth as outlined below)

### Primary outcome

Rate of cesarean delivery

### Secondary outcomes

Secondary outcomes were clinically relevant outcomes available in the dataset.

Maternal

- Third- or fourth-degree perineal laceration
- Instrumental vaginal delivery
- Diabetes in pregnancy (pre-existing type 1 or type 2 diabetes or gestational diabetes)
- Pre-term delivery
- Hypertensive disorders in pregnancy
- Induction of labour
- Augmentation of labour
- Chorioamnionitis in labour
- Unplanned hysterectomy
- Blood transfusion
- Admission to intensive care unit (ICU)
- Trial of labour

Neonatal

- Birth weight >90th percentile
- Birth weight <10th percentile
- Apgar Score < 6 at 5 min
- Assisted Ventilation immediately following delivery
- Assisted ventilation required for more than 6 h
- Neonatal intensive care unit (NICU) admission
- Antibiotics received for suspected neonatal sepsis
- Seizure or serious neurological dysfunction
- Infant breastfed at discharge

### Data collection and methods

Publicly available data from the United States Centers for Disease Control and Prevention (CDC) will be used and will be downloaded from https://www.cdc.gov/nchs/data_access/vitalstatsonline.htm[18].

Variables that are a planned part of the analysis will be interrogated to describe any missingness and extreme outliers.

### Variables

Mean and standard deviation (SD) will be used to describe continuous variables if they are normally distributed, otherwise median, and interquartile range (IQR) will be used. Categorical variables will be reported as numbers and percentages.

Gestational BMI gain in kgm^-2^ will be reported in the primary analysis. GWG in kg will be analysed for the sensitivity analysis.

Variables will be measured/described as follows:

- Gestational BMI gain in kgm^-2^ (for the main analysis). Analysed as a linear spline with a knot at 5 kgm^-2^ based on preliminary analysis of 2022 data.
- Self-reported pre-pregnancy BMI in kgm^-2^ (based on height and self-reported pre-pregnancy weight) classified according to the Institute of Medicine (IOM) groups [15]
- Measured BMI at birth in kgm^-2^ classified into groups of the same size as pre-pregnancy BMI to account for expected weight gain during pregnancy
- GWG in kg (for the sensitivity analysis)
- Parity (categorized as nulliparous, or parous)
- Maternal age in years (as a continuous variable, with linearity observed in the preliminary analysis of the 2022 data)
- Gestational age at birth in weeks (categorized by gestational week)
- Maternal height in cm (as a continuous variable, with linearity observed in the preliminary analysis of the 2022 data)
- Maternal race (to be categorized as the dataset allows)
- Maternal cigarette smoking during pregnancy (to be categorized as the dataset allows)
- Payment type (public/private)
- Trimester of onset of maternity care (first/second/third trimester/no antenatal care)
- Maternal education (categorized as (i) 8th grade or less, (ii) 9th-12th grade, no diploma, (iii) completed high school or GED, some college credit or associate degree, (iv) bachelor’s degree, (v) masters degree, doctorate or professional degree)
- Any fertility treatment used in conception (yes, no, unknown)
- Diabetes in pregnancy (to be categorized as the dataset allows)
- Hypertensive disorders in pregnancy (to be categorized as the dataset allows)
- Previous preterm birth (yes, no)

### Sample size

To estimate R^2^, pre-pregnancy BMI group and BMI gain were regressed on the other explanatory variables using equivalent US birth data for the preceding year (2022). As pre-pregnancy BMI was categorized according to IOM groups, dummy variables were created with normal BMI (18.5 to < 25) as the referent category, and each of these was regressed on the remaining dummy variables, the two BMI gain spline variables, and the other explanatory variables, resulting in five R^2^ estimations. As BMI gain was a linear spline with one knot, the two BMI gain spline terms were regressed on the five BMI group dummy variables, the remaining BMI gain spline variable, and the other explanatory variables, resulting in an additional two R^2^ estimations. These R^2^ estimations were repeated for nulliparous women only, resulting in 14 R^2^ estimations in total.

We specified an alpha value of 0.05 and beta value of 0.10, and a cesarean section rate of 18.17 % at the mean gestational BMI gain based on US birth data for 2022. This cesarean section rate was 27.40 % for nulliparous women only. We aimed to detect an increase in the cesarean section rate of one percentage point for each BMI category compared with the referent category (18.5 to < 25) (e.g., relative risk 19.17 %/18.17 %), and an increase in the cesarean section rate of one percentage point per kgm^-2^ of BMI gain. R^2^ values were used to calculate the variance inflation factor using the method described Signorini (1991) for calculating the sample size based on an exact Poisson Regression [19]. These calculations were performed using the pwrss package (Version 0.3.1) in R (Version 4.4.0).

The largest sample sizes required were:

• For the full sample including nulliparous and parous women: required sample size = 570,014
• For nulliparous women only: required sample size = 716,392

Based on the numbers of births in the 2022 data, we expect there will be enough births in the 2023 data to meet these required sample sizes

### Analysis plan

Data will be downloaded from the CDC website (https://www.cdc.gov/nchs/data_access/vitalstatsonline.htm) for the most recent year with available data (2023 at the time of writing this protocol).

If data missingness for one or more explanatory or outcome variables occurs in > 5 % and < 40 % eligible births then multiple imputation will be considered. Potential mechanisms of missingness and the relationship of missingness to the outcome and covariates will be assessed when deciding to conduct multiple imputation or a complete case analysis.

If multiple imputation is required, we will use fully conditional specification with five imputed datasets. Any multiple imputation will include all explanatory and outcome variables, and any interaction terms. Interdependencies amongst variables will be handled with the passive imputation method. P-values for the likelihood ratio test for each variable in each imputed dataset will be pooled using the median p-value rule.

Using Robust Poisson Regression, maternal pre-pregnancy BMI will be modelled on the primary outcome of cesarean delivery and other covariates (listed below) excluding BMI gain during pregnancy. Maternal BMI at the time of delivery will be modelled on the same covariates. Of these two models, the model with the lowest Akaike Information Criterion (AIC) will be used to select the BMI variable with the greatest explanatory power to be used in subsequent analyses (either pre-pregnancy or delivery BMI). This variable is referred to as ‘Maternal BMI’ in all subsequent analyses below. The two models will also be compared using the log-likelihood test.

Pre-pregnancy BMI will be categorized according to the United States Centers for Disease Control and Prevention which classifies adult BMI as underweight (BMI < 18.5), normal weight (BMI 18.5 – 24.9), overweight (BMI 25-19.9), Class 1 obesity (BMI 30-34.9), Class 2 Obesity (BMI 35-39.9), and Class 3 Obesity (BMI 40 or more) [20]. Delivery BMI will be classified such that the same numbers of women will be in each class (i.e., the same quantile cut-offs will be used for pre-pregnancy and delivery BMI groups). This is expected to account for weight gain in pregnancy including factors such as fetal weight and amniotic fluid volume. Based on the exploratory analysis for 2022 data, BMI gain and gestational weight gain will be treated as linear splines with one knot placed at 5 kgm^-2^ and at 12 kg respectively.

As nulliparous women are an important group who contribute to the first/primary cesarean delivery rate, we will test for interaction (effect modification) between maternal BMI gain and parity, and maternal BMI and parity. If interaction is present (BMI or BMI gain have a different association with cesarean delivery depending on parity), then the primary analysis will be restricted to nulliparous women only and we will conduct secondary analyses for parous women.

For the primary outcome of cesarean delivery, backward stepwise Robust Poisson Regression will be used. The following variables will be included in the multivariable Poisson regression model as potential predictors of cesarean section:

• Maternal BMI (not eligible for exclusion in the backwards stepwise regression).
• Gestational BMI gain (not eligible for exclusion in the backwards stepwise regression)
• Parity
• Maternal age (continuous)
• Gestational age
• Maternal height
• Maternal race
• Cigarette smoking
• Payment type
• Trimester of onset of maternity care (4 categories: first, second or third trimester, or no
• antenatal care)
• Education (categorized)
• Fertility treatment used
• Diabetes (3 categories: pre-pregnancy, gestational, none)
• Hypertension in pregnancy (dichotomous)
• Interaction terms: BMI x parity and BMI gain x parity

Birthweight will not be included as an explanatory variable because it was considered that fetal weight may be on the causal pathway between BMI or BMI gain and cesarean delivery. Therefore, the relative risks for BMI or BMI gain for the primary outcome of cesarean delivery will include any indirect effect mediated through fetal weight.

If Robust Poisson Regression Models do not converge then alternative regression methods will be considered.

For secondary outcomes, all explanatory variables will be retained in the model. Stepwise backward regression will not be used. No interaction terms will be used.

For the secondary outcome of third or fourth degree perineal laceration, the data will be limited to vaginal births only and the following covariates will be used: BMI at delivery, BMI gain, parity, maternal age, gestational age, maternal height, maternal race, mode of delivery (unassisted/vacuum/forceps).

For the secondary outcome of trial of labour, the following covariates will be used: BMI at delivery, BMI gain, parity, maternal age, gestational age, maternal height, maternal race, payment type, education, trimester of onset of maternity care, use of ARTs, and gestational diabetes.

For the secondary outcome of preterm birth, the same covariates will be used as for the primary outcome except gestational age will be removed and previous preterm birth will be added.

For the secondary outcome of NICU admission, the same covariates will be used as for the primary outcome of cesarean delivery.

For the uncommon or rare secondary outcomes of unplanned hysterectomy, maternal admission to the ICU, 5 min Apgar Score < 4, assisted ventilation immediately following delivery and for more than six hours, antibiotics for suspected neonatal sepsis, and neonatal seizure or serious neurological dysfunction, the same covariates will be used as for the primary outcome, if there are at least five events per variable. If there are fewer than five events per variable, then we will preselect a smaller group of the most relevant covariates or conduct a univariable analysis.

#### Subgroup analyses

A subgroup analysis will be conducted on those mothers undergoing a trial of labour, excluding those who had a planned pre-labour cesarean delivery. Parity interaction terms will be omitted (and if they were significant in the main analysis, only nulliparous mothers will be analysed, as was done for the main analysis). Stepwise backward elimination will be conducted from the beginning, including either pre-pregnancy BMI or delivery BMI, whichever was chosen in the main analysis based on predictive value there.

#### Sensitivity analysis

(1) The primary analysis will be replicated using gestational weight gain instead of gestational BMI gain.
(2) If multiple imputation is used, then the final multivariate fitted model from the imputed datasets will be replicated in the complete data for comparison.

We will report the results for the sensitivity analysis in pounds as well as kg.

## Protocol validation

We conducted an exploratory analysis of the US birth data for 2022 to inform modelling decisions. Linearity was tested for continuous variables by plotting variable quantiles against the natural logarithm of the cesarean section rate, with Wilson confidence intervals. Based on these analyses, maternal height and maternal age were linearly associated with cesarean section and will be modelled in natural units.

## Limitations

Limitations include the retrospective study design using administrative data, limiting the available variables. Misclassification and under-reporting of complications are potential concerns, although caesarean section is unlikely to be missed by reporting clinicians and the birth certificate worksheet. We have no reason to suspect any mis recording of maternal weight and height but this is possible. Maternal pre-pregnancy weight is self- reported, but we do not expect any under or over reporting of pre-pregnancy weight to be related to the primary outcome of cesearean delivery.

## Uniqueness of the study

This study is unique in that it will explore whether it is BMI rather than GWG that is linked to cesarean birth and other outcomes. Given the limited efficacy of interventions during pregnancy in modifying GWG, the study will be able to examine how BMI influences outcomes and possibly acts through mechanisms independent of GWG. This has the potential to add evidence that challenges the foundational assumptions underpinning much of the existing literature on GWG. While promoting healthy nutrition and physical activity during pregnancy remains beneficial, the study may support a better allocation of health care resources and reduce unrealistic expectations in women with regard to limiting GWG.

## CRediT author statement

Kirsten Black: Conceptualisation, methodology, write up

Owen Streeter: Conceptualisation, methodology

Arianne Sweeting: Conceptualization, methodology, manuscript review

Pejman Adily: Conceptualization, methodology, manuscript review

Mark Lauer: Conceptualization, methodology, manuscript review

Adrienne Gordon: Conceptualization, methodology, manuscript review

Vincenzo Berghella: Conceptualization, methodology, manuscript review

Belinda R Bruce: Conceptualization, methodology, manuscript review

Lauren Ferris: Conceptualization, methodology, manuscript review

Bradley de Vries: Conceptualization, methodology, manuscript review

## Declaration of competing interest

The authors declare that they have no known competing financial interests or personal relationships that could have appeared to influence the work reported in this paper.

## Data Availability

Data will be made available on request.
